# Antibacterial and Antioxidant Activities, Toxicity, and Physicochemical Properties of *Crassocephalum montuosum* (S Moore) Milne-Redh and *Crassocephalum picridifolium* (DC) S Moore

**DOI:** 10.1155/adpp/9954073

**Published:** 2024-11-28

**Authors:** Bashige Chiribagula Valentin, Bakari Amuri Salvius, Lumbu Simbi Jean Baptiste

**Affiliations:** ^1^Department of Pharmacology, Laboratory of Therapeutic Chemistry and Analysis of Natural Substances, Faculty of Pharmaceutical Sciences, University of Lubumbashi (UNILU), 27, av. Kato, Commune of Kampemba, Lubumbashi, Democratic Republic of the Congo; ^2^Department of Pharmacology, Laboratory of Pharmacognosy, Faculty of Pharmaceutical Sciences, University of Lubumbashi (UNILU), 27, av Kato, Commune of Kampemba, Lubumbashi, Democratic Republic of the Congo; ^3^Department of Chemistry, Faculty of Sciences–University of Lubumbashi (UNILU), N°1 Maternity Avenue, Commune of Lubumbashi, Lubumbashi, Democratic Republic of the Congo

**Keywords:** asteraceae, *Cavia porcellus*, DPPH, *E. coli*., gastroenteritis, *S. typhi*

## Abstract

In traditional Congolese medicine, the plants *Crassocephalum montuosum* (CrasMon) and *Crassocephalum picridifolium* (CrasPic) are used to treat bacterial gastroenteritis. In the present study, the antibacterial and antioxidant activities as well as the acute and subacute toxicity of organic extracts from the whole plant of the two investigated taxa were evaluated. Physicochemical parameters were also determined, and total phenolics, flavonoids, and tannins were investigated and assayed. The antibacterial and antioxidant activities of the plant extracts were evaluated using disc diffusion, tube macrodilution, and DPPH tests. Conversely, traditional solution reactions, gravimetric tests, and spectrophotometric tests were used to generate physicochemical profiles, identify secondary metabolite groups, and perform microdilution and DPPH tests to evaluate the antibacterial and antioxidant activities, respectively. OECD tests were adapted to assess the acute and subacute toxicity. All the extracts showed antibacterial activity against *E. coli* and *S. typhi* strains with the diameter zone of inhibition (DZI) ranging from 12 to 23 mm and the minimum inhibitory concentration (MIC): 15.625–125 μg·mL^−1^. The methanolic extract of CrasPic showed the most pronounced activity with a DZI of 21–23 mm and MIC of 15.625–62.5 μg·mL^−1^. All extracts showed high antioxidant activity with IC_50_ (half maximal inhibitory concentration) ranging from 11.6 to 21.8 μg·mL^−1^, with the methanolic extract of CrasMon showing the most pronounced activity. Both plants contain a variety of phytochemicals including coumarins, quinones, flavonoids, phenols, saponins, tannins, and terpenoids. The methanolic extract of CrasPic exhibits the highest content of total phenolics (300 mg·GAE·g^−1^), flavonoids (56 mg·QE·g^−1^), and tannins (155 mg·GAE·g^−1^). These extracts have a median lethal dose (LD_50_) > 5000 mg·kg^−1^ and no signs of toxicity at 200 mg·kg^−1^ after 30 days of oral administration to *Cavia porcellus*. The total ash content was determined to be 14.2% and 15.8% (on a dry weight basis), with the ash insoluble in hydrochloric acid exhibiting a range of 4.04%–5.03%. CrasMon and CrasPic have been demonstrated to exhibit a good antibacterial and antioxidant activities, at least in part, due to the presence of phenolic compounds. These activities may provide a rationale for their use in traditional Congolese medicine against gastroenteritis.

**Summary**

1. Crassocephalum picridifolium (CrasPic) and *Crassocephalum montuosum* (CrasMon) are used in traditional medicine in Lubumbashi for the treatment of gastroenteritis without any evidence of efficacy in the accessible literature.
2. CrasPic has the best antibacterial activity on all germs tested, and CrasMon has the best antioxidant activity.
3. CrasPic exhibits the highest levels of total phenols, flavonoids, and tannins. Nevertheless, both taxa demonstrate an excellent linear correlation between antibacterial and antioxidant activities and total phenols, flavonoids, and tannins.
4. Both taxa were practically nontoxic in vivo to *Cavia porcellus* L with LD_50_ > 5000 mg·kg^−1^.

## 1. Introduction

*Crassocephalum montuosum* (S. Moore) Milne-Redh (CrasMon) and *Crassocephalum picridifolium* (DC.) S. Moore (CrasPic) are two species of the Asteraceae family endemic to sub-Saharan Africa. Both species are used in traditional Congolese medicine to treat chronic gastroenteritis [1].

CrasMon, locally known as Cifula (Shi) or Bupamba (Bembe), contains coumarins, flavonoids, quinones, and terpenoids [2]. Many terpenoids have been identified within the plant, with *α*-phellandrene (87.6%) and *β*-caryophyllene (0.2%) representing the majority [3]. A previous study [4] demonstrated that the plant exhibited antiradical properties. In addition to its use in managing acute gastroenteritis, this taxon is employed in wound management in the Democratic Republic of the Congo (DRC). Moreover, the plant is utilized in the treatment of amebiasis, diarrhea, hernia, placental retention, malaria, nutritional, and skin disorders [1, 5]. In other African countries, the plant is also employed for therapeutic applications, including wound care, ant repellents, and diarrhea in Uganda [6–8], female infertility in Kenya [9], and ear ache, headache, and burn in Tanzania [10]. In Angola, the plant is used as a wild food [11]. In Burundi, the specie is involved in livestock [12]. To the best of our knowledge, no studies evaluating the antibacterial activity have been reported in the accessible literature.

In contrast to CrasMon, CrasPic, locally known as Mfuburdi (Bembe), needs to be better documented in the available literature. The plant is employed in Kenya to treat gastrointestinal ulcers [9]. In the DRC, the plant treats influenza, cataract eye, goiter, stomach pain, diabetes, hemorrhoids, gonorrhea, hepatitis, and otitis [1, 13]. An earlier study identified the antioxidant activity and the presence of coumarins, flavonoids, quinones, and terpenoids in the whole plant [2].

While antibiotic therapy has undoubtedly played a significant role in the fight against bacterial infections, it is also essential to acknowledge that antibiotics have been used on a massive scale, leading to the emergence of resistance [14, 15]. This resistance phenomenon is prompting researchers to explore alternative approaches to combating infections. One approach that is increasingly being utilized is the ethnopharmacological approach. This approach involves examining natural substances that local populations have utilized for an extended period. The utilization of ethnomedicinal knowledge is constrained by the paucity of knowledge regarding the efficacy and safety of these natural resources. This study is situated within the broader context of previous research that has screened natural resources, particularly medicinal plants, for the development of new antimicrobial [16, 17] and antioxidant [18–20] recipes, as well as the assessment of their toxicities [21–23].

The objective of this study was to evaluate the in vitro antimicrobial effectiveness of methanolic and ether extracts (EEs) of CrasPic and CrasMon, whole plants, against bacterial species frequently associated with bacterial gastroenteritis. Moreover, the objective was to assess their potential antioxidant activity and acute and subacute toxicity. In addition, the physicochemical parameters were determined, and the total phenols, flavonoids, and tannins were investigated and assayed.

## 2. Materials and Methods

### 2.1. Plant Material and Animal Model

The plant material used in this study consisted of whole plants of CrasMon (11°35′26.00052″S; 27°26′18.21552″E; 1264.2 m) and CrasPic (11°34′24″S; 27°22′43″E; 257.5 m). Whole plants are used in traditional medicine to treat gastro-enteritis. The taxa were collected in May 2022 in Lubumbashi, DRC, and identified at the Kipopo herbarium (coupon numbers: CrasMon, KIP437629967; CrasPic, KIP437629964).

The *Cavia porcellus* specimens (382.5 ± 4.2 g) utilized as animal models were procured from the animal holding unit of the Zoo Technology Department, Faculty of Agronomic Sciences, University of Lubumbashi. Throughout the experiments, the animals were provided with unlimited access to local rodent food (MIDEMA-RDC) and water *ad libitum*. The animals were housed in groups in metal cages (5 × 5 × 5 m^3^) and maintained in a standard laboratory environment.

### 2.2. Reagents, Substrates, and Solvents

The reagents, substrates, and solvents utilized in the experiments included ascorbic acid, 1,1-diphenyl-2-picrylhydrazyl radical (DPPH), ciprofloxacin, gallic acid, methanol, quercetin, and vanillin, which were obtained from Sigma-Aldrich (USA). All chemicals and solvents employed were of analytical quality.

### 2.3. Extract Preparations

The petroleum EEs were obtained via the maceration of 350 g of coarse, powdered, and air-dried plant material in 1.5 L of petroleum ether over a period of 72 h. Subsequently, the extractable matter was filtered on three occasions, each with the same solvent, resulting in a yield of 16.2% and 3.16%, respectively (m/m). The pomace was then extracted with methanol for a period of 72 h. Following the maceration process, the extract was filtered through Whatman paper (USA), and the pomace was extracted twice with methanol. The resulting extractable matter was found to be 30.2% and 21.5% (m/m), respectively. The filtrates of the same solvents were combined, concentrated, and dried using a rotary evaporator (Büchi R-210, Switzerland) at 40°C under reduced pressure (130–180 mbar). For all tests, each extract was dissolved in its extraction solvent prior to combination with the vehicle.

### 2.4. Antibacterial Activity Test

The antibacterial activity was assessed by the macrodilution test in a tube, by determining MIC, and by macrodiffusion on a disk, by determining the DZI [21, 24, 25].

The bacterial strains included in this study were *Escherichia coli* ATCC (American Type Culture Collection) 25,922 (*E. coli*), *Campylobacter jejuni* ATCC 33291 (*C. jejuni*), *Staphylococcus aureus* ATCC 6538 (*S. aureus*), *Shigella sonnei* ATCC 25931 (*S. sonnei*), and *Salmonella typhi* ATCC 14028 (*S. typhi*). The bacterial strains were obtained from the Lubumbashi Provincial Laboratory, where tests were conducted using an agar diffusion method to ascertain the diameter of the zone of inhibition (DZI). Additionally, the broth dilution method was employed in triplicate to ascertain the minimum inhibitory concentration (MIC) and the minimum bactericidal concentration (MBC) of the extract [20].

These distinct parameters (DZI, MIC, and MBC) were utilized to delineate the antimicrobial activity of the extracts. The microorganisms selected for this study were based on their prevalence in locally observed instances of bacterial gastroenteritis.

For susceptibility testing, 20 mL of Muller-Hinton agar was poured into sterile Petri dishes and allowed to stand for 20 min. Subsequently, 1 mL of a bacterial suspension containing 10^8^ CFU (colony-forming units) per mL was evenly distributed over the entire surface of each culture medium. Blotting paper discs (with a diameter of 6 mm) were impregnated with 5 μL of extracts (with a concentration of 50 μg·mL^−1^) and placed on the surface of the solidified, infected medium. Subsequently, the Petri dishes were incubated at 37°C for 48 h in an oven. The susceptibility of the germs to the extracts was estimated by measuring the DZI (in millimeters) induced by different concentrations of plant extracts around the discs. Each experiment was repeated three times. The extracts were categorized as follows according to their MIC values: very active (MIC < 100 μg/mL), moderately active (100 ≤ MIC < 500 μg/mL), weakly active (500 ≤ MIC ≤ 1000 μg/mL), and inactive (MIC > 1000 μg/mL) [21, 24].

To determine MIC, 100 μL of each extract at a concentration of 1 mg/mL, dissolved in methanol (petroleum ether), was combined with 1900 μL of culture medium. Subsequently, eight successive dilutions of order 2 (ranging from 500 μg·mL^−1^–1.9 μg·mL^−1^) were prepared for each extract and placed in different 5 mL aseptic tubes. After that, 1000 μL of standard inoculum was added to each tube, and the mixture was incubated for 24 h at 37°C. The growth of microorganisms was observed visually. The MIC was determined as the lowest concentration at which the extract prevented visible bacterial growth [26]. Extracts were categorized as follows according to their MIC values: very active (MIC < 100 μg·mL^−1^), moderately active (100 ≤ MIC < 500 μg·mL^−1^), weakly active (500 ≤ MIC ≤ 1000 μg·mL^−1^), and inactive (MIC > 1000 μg·mL^−1^).

To determine the MBC, samples were taken from tubes that exhibited no visible growth, as had been used to determine the MIC. Inoculation was conducted in Petri dishes on Mueller–Hinton agar medium, and incubation was performed at 37°C for 24 h. The growth of microorganisms was monitored visually, and the MBC, defined as the lowest concentration at which the extract prevented visible bacterial growth after transplantation, was determined [27]. The effect of the extract was determined according to the ratio between the MBC and the MIC. A bactericidal effect was considered present when the ratio was ≤ 2, while a bacteriostatic effect was observed when the ratio was > 2 [21]. The experiment was performed in triplicate.

### 2.5. DPPH Assay

The antioxidant activity of the samples was evaluated using the DPPH assay, as previously described [28, 29]. Briefly, 50 μL of extracts (or positive control) was prepared at different dilutions of order two in methanol from 100 g·mL^−1^ solution and was interacted with 1950 μL of 0.002% DPPH in a 96-well plate (Nunc WVR, Germany). Following a 30-min incubation period in the dark, the solution was read at 492 nm (Thermo Fisher Scientific Inc., Waltham, USA). The assays were conducted in triplicate, with the 0.002% DPPH solution as the negative control. The percentage of antioxidant activity was calculated using the following formula:

(1) $$\begin{array}{c} \text{AAO}\left(\%\right)=\frac{\text{Ab}-\text{Ae}}{\text{Ab}}\times 100\%, \end{array}$$

where Ab is the absorbance measured in the presence of the negative control, Ae is the absorbance measured in the presence of the extract, and AAO is the calculated percentage of inhibition. The experiment was performed in triplicate.

In this study, the antioxidant activity of the extracts was categorized as follows: very strong when IC_50_ < 50 μg/mL, strong when 50 μg/mL ≤ IC_50_ ≤ 100 μg/mL, moderate when 100 μg/mL < IC_50_ < 250 μg/mL, weak when 250 μg/mL < IC_50_ < 500 μg/mL, and absent when IC_50_ ≥ 500 μg/mL [28, 30].

### 2.6. Toxicity Tests

Acute toxicity was assessed as previously described [23] by administration of a single oral dose of 5000 mg·kg^−1^ of extracts by gavage (test groups). Animals (5 per group) were observed for 28 days, clinical signs were observed daily, and body weights were measured every 7 days. On the last day, blood was collected from the jugular vein and serum was prepared for analysis of biochemical parameters (AST, ALT, ALP, urea, and creatinine). For the determination of the 5000 mg·kg^−1^ dose, we took into account the preliminary studies of the acute toxicity assessment according to the Organization for Economic Co-operation and Development (OECD) method, in which no signs of toxicity were observed up to 3000 mg·kg^−1^.

In the subacute test, *Cavia porcellus* (5 animals per group) were given 200 mg·kg^−1^ bw per day (test group). In this subacute toxicity study, in addition to the biochemical parameters examined in the acute toxicity evaluation, the weights of the valuable organs (liver, heart, rate, and kidney) were weighed after the animals were sacrificed on day 28. The choice of dose for the assessment of subacute toxicity is in line with the approximate dose used in traditional Congolese medicine [1].

Validated procedures were followed for blood collection and serum preparation for biochemical analysis [31]. Alkaline phosphatase (ALP), aspartate aminotransferase (AST), alanine aminotransferase (ALT) activities, and urea and creatinine levels were determined by colorimetric assays using Labtest® kits (Minas Gerais, Brazil).

### 2.7. Preliminary Phytochemical Screening

The whole plant powders were analyzed for the presence of alkaloids, coumarins, flavonoids, saponins, steroids, tannins, terpenoids, and phenols. This was conducted using standard tube reactions, as previously reported [2, 4, 32].

### 2.8. Determination of Total Phenol, Flavonoid, and Tannin Content

The total phenolic content of each sample was quantified using a Folin–Ciocalteu method [33]. The procedure involved the preparation of a solution of extract (100 μg·mL^−1^) or gallic acid (100–600 μg·mL^−1^) in 1 mL of 10% (w/v) Folin–Ciocalteu reagent, which was then mixed with 2.5 mL of the solution. Subsequently, 2.0 mL of Na_2_CO_3_ (75%) was added to the mixture, which was then incubated at 50°C for 10 min with intermittent stirring. Subsequently, the sample was cooled, and the absorbance was measured using a UV spectrophotometer (Shimazu, UV-1800) at 765 nm against a blank. The results were expressed in milligrams of gallic acid equivalents per gram of dry extract (mg·GAE·g^−1^) using a calibration curve (*y* = 0.0699*x* + 0.0127, *R*^2^ = 0.998; linearity range, 1–1200 mg·mL^−1^).

As previously described, the total flavonoid content (TFC) was quantified using an aluminum trichloride assay [34]. Briefly, an aliquot of 1 mL extract solution (100 μg·mL^−1^) or quercetin (25–250 μg·mL^−1^) was combined with 0.2 mL of a 10% (w/v) AlCl_3_ solution in methanol, 0.2 mL of a 1 M potassium acetate solution, and 5.6 mL of distilled water. The mixture was incubated for 30 min at room temperature, after which the absorbance was measured at 415 nm against the blank. The results were expressed in milligrams of quercetin equivalents per gram of dry extract (mg·QE·g^−1^) using a quercetin calibration curve (*y* = 0.006 *x* + 0.003, *R*^2^ = 0.996; linearity range, 0.1–150 mg·mL^−1^).

The total condensed tannin content was determined using a vanillin method, as previously described [35]. Briefly, 1 mL of a solution of the extract (100 μg/mL) or gallic acid (100–600 μg/mL) was mixed with 5 mL of a vanillin solution (0.5 g reagent and 200 mL 4% HCl in methanol). The absorbance at 500 nm, following a 20-min reaction time, was quantified using a spectrophotometer (SP 2000 UV, Bel Photonics®, Piracicaba, SP, Brazil) in comparison to a blank. The results were expressed in milligrams of gallic acid equivalents per gram of plant dry extract (mg·GAE·g^−1^ED) using a calibration curve based on gallic acid (*y* = 0.004 *x* + 0.0013, *R*^2^ = 0.997; linearity range, 1–100 mg·L^−1^).

### 2.9. Determination of Physicochemical Parameters

#### 2.9.1. Loss on Drying (LOD)

The LOD was quantified according to the following protocol: A flat-bottomed capsule with a diameter of approximately 50 mm and a height of approximately 30 mm was used to weigh 0.50 g of finely pulverized dry drug. The capsule was then oven-dried at 105°C for 3 h. After drying, the capsule was cooled in a desiccator, and the weight was recorded. The result was expressed as a percentage:

(2) $$\begin{array}{c} \text{LOD}\left(\%\right)=\frac{a}{b}\times 100\%, \end{array}$$

where *a* is the weight after proofing, and *b* is the weight of the sample before proofing.

#### 2.9.2. Total Ash (TA)

One gram of the pulverized drug was placed in a porcelain crucible. The test sample was distributed evenly within the crucible. The sample was subjected to an oven drying process for one hour at a temperature range of 100°C–105°C. After that, it was incinerated in a muffle furnace at 600 ± 25°C. It is important that the sample does not ignite at any point during this process. Once the sample had been incinerated, it was allowed to cool in a desiccator before being weighed using an analytical balance. Subsequently, the crucible was permitted to cool in a desiccator before being weighed on an analytical balance. The TA content was calculated as follows:

(3) $$\begin{array}{c} \text{TA }\left(\%\right)=\frac{m}{n}\;x\;100\;\%, \end{array}$$

where *m* is the weight after incineration and *n* is the test sample.

#### 2.9.3. Hydrochloric Acid Soluble Ash (HAA)

To ascertain the ash content, 15 mL of distilled water and 10 mL of hydrochloric acid were added to the residue obtained during the TA determination. Subsequently, the crucible was covered with a watch glass and boiled gently for 10 minutes. Following this, the crucible was allowed to cool. Subsequently, the residue was filtered through an ash-free filter paper, with the filtrate subsequently washed with hot water until it reached a neutral pH. Subsequently, the filtrate was transferred to a dry, tared crucible. Subsequently, the crucible containing the filter paper was subjected to an oven firing for 24 h. Subsequently, the crucible was subjected to a further calcination process for 6 hours at a temperature of 600°C. Following cooling in a desiccator, the crucible containing the ash was reweighed, and the hydrochloric ash was calculated as follows [36]:

(4) $$\begin{array}{c} \text{HAA}\left(\%\right)=\frac{P1}{P2}\times 100\%, \end{array}$$

where *P*1 is the weight after calcination and *P*2 is the weight before calcination.

#### 2.9.4. Swelling Index

The swelling index is defined as the volume in milliliters occupied by 1 g of drug. The volume of the graduated cylinder should be 25 mL, with graduations of 0.5 mL and a height of 125 ± 5 mm (diameter 1.5 cm). The introduction of 1.0 g of the drug in its whole or powdered form is required. The drug should be moistened with 1.0 mL of 90° alcohol, and then, 25 mL of distilled water should be added. The test tube should be stopped. The sample should be vigorously shaken every 10 min for 1 hour, after which it should be allowed to stand for 3 hours. The volume occupied by the drug should then be measured. Three tests should be conducted simultaneously. The swelling index is calculated as the mean of the three tests.

### 2.10. Statistical Analysis

The data were analyzed using GraphPad Prism 6 (GraphPad Software, La Jolla, USA). Comparisons between the different groups were made by analysis of variance (ANOVA), with a probability level of *p* < 0.05 considered significant.

### 2.11. Ethical Considerations

The principles governing the use of laboratory animals as set out by the OECD, the University of Montreal's Animal Use Ethics Committee, and the Canadian Council on Animal Care protocol were duly observed. The project proposal and procedures were reviewed and approved by the Department of Pharmacology, Faculty of Pharmaceutical Sciences, University of Lubumbashi, DRC (FSP-UNILU-DP-BD-032023).

## 3. Results

### 3.1. Sensitivity of Bacteria to Different Extracts

The extracts showed a diameter of inhibition zone (DZI) ranging from 07.8 ± 1.2 mm (CrasMonE on *S. aureus*) to 22.9 ± 0.1 mm (CrasPicM on *E. coli*) on the different germs studied in solid medium. According to the classification system previously proposed [37], all the extracts showed activity (DZI > 10 mm) against *E. coli* and *S. typhi*. In addition, the CrasPic methanolic extract showed activity against all the microorganisms tested (Table 1).

### 3.2. MIC, MBC and Effect of Extracts

The MICs of the extracts ranged from 15.625 to 500 μg/mL. With the exception of the EE of CrasMon, all the extracts showed an MIC of 15.625 μg/mL, the lowest, against *E. coli*. In contrast to the other extracts, which showed bactericidal activity against all microorganisms tested, the EE of CrasMon showed a bacteriostatic effect on *E. coli*, *C. jejuni* and *S. typhi*. The classification of extracts by MIC (2.4) used in this study showed that all extracts exhibited strong antibacterial activity against *E. coli* and *S. typhi* (MIC < 100 μg·mL^−1^). CrasMon showed high activity against *C. jujeni*, while CrasPic showed activity against *S. sonnei*, with its methanolic extract showing high activity against all germs tested (Table 2).

### 3.3. Antioxidant Activity

**Figure 1:**
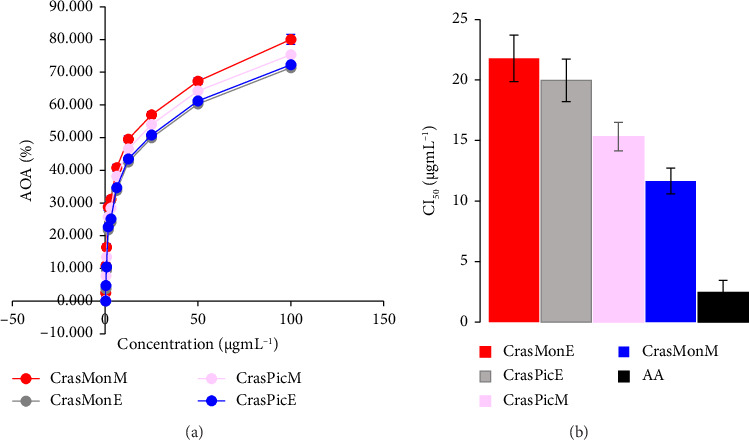
Antioxidant activity of organic extracts of *Crassocephalum montuosum* and *Crassocephalum picridifolium* expressed as percentage DPPH inhibition (a) and as IC_50_ (b); (($\overset{¯}{X\;}$) ± *S*, *n* = 3). Legend—AA: Ascorbic acid used as positive control; AOA: Antioxidant activity.

The antioxidant activity of the extracts was found to vary significantly, with values ranging from 3.4% to 100% in accordance with the concentration and exponential model employed. In accordance with the aforementioned classification (2.5), the extracts demonstrated high antioxidant activity, as evidenced by their IC_50_ values (11.6–21.8 μg·mL^−1^). However, this activity was observed to be at a lower level than that of ascorbic acid (IC_50_ = 2.46 μg·mL^−1^), which served as a positive control. The methanolic extract of CrasMon exhibited the highest antioxidant activity (Figure 1).

### 3.4. Phytochemical Groups Identified

With the exception of alkaloids, all other secondary metabolite groups (anthraquinones, coumarins, flavonoids, polyphenols, saponins, steroids, and terpenoids) were identified in the aerial parts of CrasMon and CrasPic. CrasPic demonstrated the highest number of identified phytochemical groups, with seven out of nine (Table 3).

The total phenol and tannin contents were expressed as gallic acid equivalents per gram (mg·GAE·g^−1^), and the range was observed to be from 223.3 ± 1.9 to 300.8 ± 3.1 for phenols, as well as from 85.0 ± 5.0 to 155.0 ± 5.0 for tannins, respectively. The TFC, expressed as quercetin equivalents per gram (mg·QE·g^−1^), was found to be 50.48 ± 0.44 to 56.34 ± 0.35. In general, the highest values were observed with the methanolic extract of CrasPic (Table 4).

### 3.5. Correlations Between Total Phenol, Total Flavonoid, and Antioxidant and Antibacterial Activities

**Figure 2:**
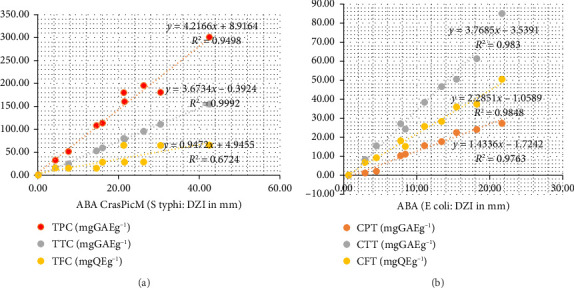
Linear correlation between antibacterial activity of CrasPic on *S. typhi* (a) and antibacterial activity of CrasMon on *E. coli* (b) with total phenolic content (red), total condensed tannin content (gray), and total flavonoid content (yellow), respectively.

**Figure 3:**
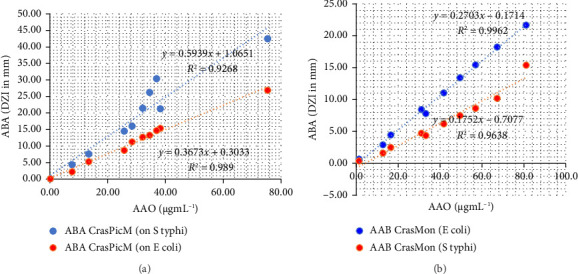
Linear correlation between antioxidant and antibacterial activities of *Crassocephalum picridifolium* (a) and *Crassocephalum montuosum* (b) methanolic extracts.

A positive linear correlation (*R*: 0.934–0.999) was observed between the antibacterial activity of CrasPic on *S. typhi* and the total phenolic content (*y* = 6.9074*x* + 4.9152; *R* = 0.97) and total tannins content (*R* = 0.999). Similarly, the antibacterial activity of the EE of CrasMon on *E. coli* exhibited a positive linear relationship (*R*: 0.97–0.98) with the content of total phenolics, flavonoids, and tannins (Figure 2(b)). Furthermore, a positive linear relationship was identified between antioxidant and antibacterial activities for the two taxa on both strains, *E. coli* and *S. typhi* (Figure 3).

### 3.6. Toxicity In Vivo

**Figure 4:**
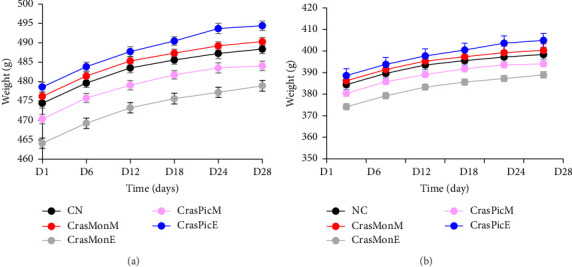
Weight evolution of *Cavia porcellus* during acute (a) and subacute (b) toxicity evaluation after oral administration of 5000 mg·kg^−1^ (a) and 200 mg·kg^−1^ (b) methanolic (M) and etheric (E) extracts of *Crassocephalum montuosum* (CrasPic) and *Crassocephalum picridifolium* (CrasPic) leaves (($\overset{¯}{X\;}$) ± *S*, *n* = 3).

**Figure 5:**
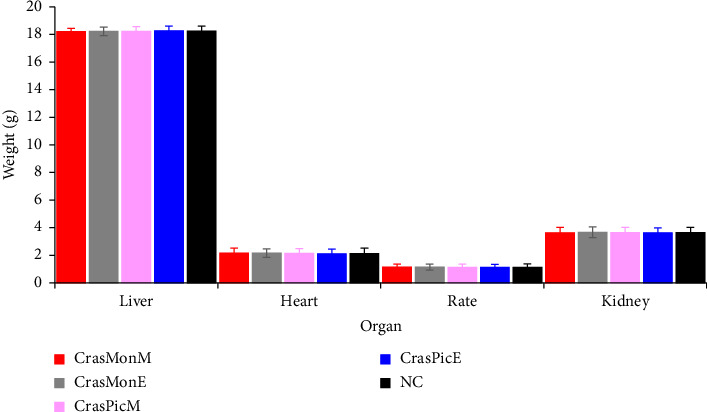
Variation in organ weights of *Cavia porcellus* when assessing the subacute toxicity of methanolic (M) and ether (E) extracts of the aerial parts of *Crassocephalum montuosum* (CrasPic) and *Crassocephalum picridifolium* (CrasPic) (($\overset{¯}{X\;}$) ± *S*, *n* = 3).

The acute toxicity of the extracts was evaluated through the administration of a single oral dose of 5000 mg·kg^−1^ body weight to guinea pigs. The results demonstrated that the extract did not induce mortality or alterations in body weight when compared to the control group (Figure 4(a)). No motor, respiratory, or cardiac disturbances were observed. Furthermore, when subacute toxicity was assessed following the administration of 200 mg·kg^−1^, no variation in body weight (Figure 4(b)) or organ weights was observed (Figure 5).

Similarly, no variation in hepatic, cardiac, or renal function biomarkers was observed between the standard control group and the test groups (Table 5).

### 3.7. Physicochemical Characteristics

The TA of these two taxa exhibits a range of 14–15%, while the hydrochloric ash represents approximately one-third of the TA. The extractable matter by methanolic extract varies between 30% and 33%, while that extractable by petroleum ether varies between 21% and 22%. The LOD is less than 1%, and the swelling index is less than 6 mL. The pH of a 10% (w/v) solution ranges from 5.4 to 6.7 mol/L (Table 6).

## 4. Discussion

Bacterial gastroenteritis is a common reason for seeking traditional medicine or self-medication with plants in Lubumbashi. However, there is a lack of evidence to support the efficacy or safety of these practices. This study focused on two plants of the Crassocephalum genus, CrasMon and CrasPic, whose whole plant are utilized in Lubumbashi to treat gastroenteritis. This study evaluated the antibacterial activity and in vivo toxicity of the plants, establishing a correlation between antibacterial activity and the presence of groups of substances with antioxidant potential, including polyphenols, flavonoids, and tannins. Additionally, the study aimed to ascertain the physicochemical properties of the two plants.

### 4.1. Antibacterial Activity of Plant Extracts

This study reports, for the first time, the antibacterial activity of methanolic and EEs, of whole plant, of CrasMon and CrasPic on *C. jujeni* and *S. sonnei* (Tables 1 and 2), as no related activities are reported in the accessible literature.

A review of the literature reveals no reports on the antibacterial activity of the two taxa analyzed in this study. Nevertheless, at the genus level, research has primarily concentrated on *E. coli* and *S. aureus* bacteria. At a concentration of 50 μg·mL^−1^, the aqueous extract of *C. crepidioides* (Benth.) S Moore leaves exhibited antibacterial activity, with respective DZI values of 10 and 13 mm [26]. In *C. biafrae* (synonym of *Solanecio biafrae* (Oliv. & Hiern) C. Jeffrey), at 1000 μg·mL^−1^, its methanolic extract from roots demonstrated antibacterial activity against *E. coli* (DZI = 30.15 mm). In contrast, no activity was observed against *S. aureus*, with a DZI value of less than 10 mm [38]. The methanolic extracts of the two plants, CrasMon and CrasPic, demonstrated superior antibacterial activity, with DZI values exceeding 14 mm against *E. coli* and *S. aureus*, thereby outperforming the previously mentioned taxa. In contrast to the EE of *C. crepidioides*, their EEs are inactive. Prior research has indicated that *C. crepidioides* displays antibacterial activity against *E. coli*, *S. aureus*, and *S. typhi* [26, 39–41]. However, this activity is relatively low in comparison to the antibacterial activity observed in the present study, which was observed in the methanolic extracts of CrasMon and CrasPic. The antibacterial activity of *C. crepidioides* has been linked to the presence of essential oils, specifically *α*-caryophyllene and *β*-cubebene, in its leaves and thymol and 4-cyclohexybutyramide in its stem. These compounds belong to the terpenoid groups identified in the two taxa analyzed in the present study. Furthermore, the search for the compounds responsible for the antibacterial activity of these two plant taxa should be expanded to include terpenoids.

Additionally, previous studies have investigated other species of the Crassocephalum genus for their antibacterial properties against *Escherichia coli* and *Staphylococcus aureus*, with varying results. This is exemplified by the ethyl acetate extract of *Crassocephalum bauchiense* (Hutch.) Milne-Redh leaves. The MIC values for *S. aureus*, *E. coli*, and *S. typhi* were 48 and 1562 μg/mL, respectively [42]. The MIC values for the hydroethanolic extract of *Crassocephalum vitellinum* (Benth.) S. Moore aerial parts were 3.125 mg/mL [43]. The values obtained with CrasMon on *E. coli* and *S. typhi* are higher than those observed with the aforementioned taxa.

Prior research has indicated that the antibacterial efficacy of the extracts can be attributed to the disruption of membrane permeability, leakage of cellular contents, reduction of pH, and hyperpolarization of the bacterium's intracellular contents. Furthermore, the disruption of the organism's redox system has been observed, which leads to the inhibition of numerous oxygen reactions that are vital to the bacteria and thus results in a bactericidal effect [36, 44]. This latter mechanism may explain why, as observed in previous studies [45–47], a positive correlation is observed between antioxidant activity and antibacterial activity. Such a correlation can be observed, in particular, with extracts whose antibacterial activity is essentially linked to phenolic compounds. This is particularly evident in the studies previously mentioned. It should be noted, however, that neither antioxidant nor antibacterial activity is solely linked to phenolic compounds.

It is similarly conceivable that nonphenolic compounds may be the source of the observed activity. This is exemplified by carotenoids [48], limonene, linalool, and citral, which are standard nonphenolic terpenoid components of essential oils [49], or 7-dialkyl-aminocoumarin, a nonphenolic coumarin [50], or the compounds: 1-O-*β*-d-glucopyranosyl-2-methoxy-3-(2-hydroxy-triaconta-3,12-dienoate) glycerol and 3-O-[*α*-l-rhamnopyranosyl-(1-4)-*β*-d-glucopyranosyl-(1-3)]-[-d-glucopyranosyl-(1-2)]-*β*-d-fucopyranosyl-olean-11,13(18)-diene-3,23. The compound in question is 3-O-[*α*-l-rhamnopyranosyl-(1-4)-*β*-d-glucopyranosyl-(1-4)-*β*-d-glucopyranosyl-(1-3)]-*β*-d-fucopyranosyl-olean-11,13(18)-diene-3,23,28-triol. The same is true of 3-O-[*α*-l-rhamnopyranosyl-(1-4)-*β*-d-glucopyranosyl-(1-3)]-[-d-xylopyranosyl-(152)]-*β*-d-glucuronopyranosyl-acid-olean-11,13(18)-diene-3,23,28-triol [51]. The preliminary phytochemical screening of the two taxa revealed the presence of additional phytochemical groups, including terpenoids and coumarins (Table 3). It can be reasonably inferred that these may have contributed to the observed antioxidant activity, although this remains correlated with phenols. Further detailed studies are necessary to gain a comprehensive understanding of the subject matter.

### 4.2. Antioxidant Activity of Extracts

This study presents the initial evidence of the antioxidant activity of EEs derived from the CrasPic and CrasMon, whole plant (Figure 1). A previous study reported the antiradical activity of methanolic extracts of specimens of these two plants harvested in Bukavu, located in the eastern region of the DRC, during the rainy season (January and February 2014). The respective IC_50_ values were 47.7 ± 0.2 and 60.2 ± 0.2 μg·mL^−1^ [2]. The observed activity was found to be lower than that observed in the present study (IC_50_: 11.6–21.8 μg·mL^−1^) on specimens collected in Lubumbashi in southern DR Congo during the dry season. The study was conducted from May to June 2023 (Figure 1). In general, the activity of a plant is correlated with its qualitative and quantitative composition of bioactive substances, which can vary according to soil type, climate, and, in the context of traditional medicine, harvesting conditions [52–54]. However, it would be beneficial to harvest both taxa under identical conditions to determine if the differences observed are attributable to the presence of distinct cultivars. Additionally, further investigation could assess the impact of each of the aforementioned factors on this notable alteration in the free radical scavenging capacity of extracts.

It has been reported that six taxa currently accepted in the genus Crassocephalum possess free radical scavenging properties, with the two subtaxa excluded. This is particularly evident in the case of *C. vitellinum*, for which the methanolic extract was found to be active with an IC_50_ of 17.6 ± 0.8 μg·mL^−1^. The essential oil extracted from this plant contains the following significant compounds: limonene (34.8%), E-*β*-pinene (8.5%), *α*-pinene (6.6%), and myrene (6.3%). Additionally, the essential oil exhibited antioxidant activity, with an IC_50_ of 28.8 ± 2.0 μg·mL^−1^ [55]. Similarly, the ethyl acetate extract of the aerial parts of *C. bauchiense* has been demonstrated to exhibit antiradical activity, with an IC_50_ of 55.79 ± 5 μg·mL^−1^. The extract demonstrated activity with an IC_50_ of 40 μg·mL^−1^ [56]. Similarly, *Crassocephalum macropappum* (Sch Bip ex A Rich) S Moore, for which a methanolic leaf extract was found to be active with an IC_50_ of 12.4 ± 2.31 μg·mL^−1^ [57], and *C. rubens*, for which a methanolic extract is reported to be antiradical with an IC_50_ of 1.81 ± 0.21 μg·mL^−1^ [58], also demonstrated noteworthy activity. The methanolic extract of the aerial parts of *Crassocephalum bougheyanum* CD Adams demonstrated antiradical activity with an IC_50_ of 23.7 ± 1.18 μg·mL^−1^ [59]. *C. crepidioides* is also worthy of mention as a plant with antiradical properties belonging to the genus Crassocephalum, given that its leaves have been shown to be active with an IC_50_ of 65.3 μg·mL^−1^ [60]. The remaining taxa of the genus Crassocephalum, with the exception of *C. rubens*, have been demonstrated to possess antiradical activity with a potential in the range of 12–60 μg·mL^−1^. This activity is lower than that exhibited by the methanolic extract of CrasMon (IC_50_ = 11.65 ± 1.21 μg·mL^−1^), as revealed during the present study (Figure 1). It is noteworthy that *C. rubens* displays a higher antioxidant potential than the two taxa under consideration in the present study.

The two taxa, CrasMon and CrasPic, demonstrate an intriguing antioxidant potential that may contribute to their resilience in response to stress conditions. This, in turn, enhances their capacity to maintain cellular homeostasis [61]. This potential also positions them among the plants most likely to demonstrate antibacterial, anti-inflammatory, antidiabetic, and healing properties. These properties are consistent with the other ethnobotanical virtues [1] for which they are used in traditional Congolese medicine.

### 4.3. Preliminary Phytochemical Screening of CrasMon and CrasPic

From a phytochemical perspective, the presence of coumarins, flavonoids, saponins, steroids, and terpenoids in CrasPic and of saponins, tannins, and terpenoids in CrasMon (Table 3) is consistent with previous studies conducted on taxa harvested in the town of Bukavu (DRC) during the rainy season [4] whereas the presence of flavonoids in CrasMon and tannins in CrasPic differs from the study above. It is, therefore, possible that the biosynthesis of coumarins, flavonoids, saponins, steroids, and terpenoids in CrasPic and that of saponins, tannins, and terpenoids in CrasMon are independent of climatic and soil conditions, in contrast to the biosynthesis of anthraquinones and saponins in CrasPic and flavonoids in CrasMon.

For the first time, this study presents the total phenol, flavonoid, and tannin content of CrasMon and CrasPic (Table 4). At the genus level, *C. bougheyanum* exhibited total phenol content (TPC) of 61.82 ± 0.14 mg·GAE·g^−1^, total tannin content (TTC) of 9.08 ± 0.62 mg·GAE·g^−1^, and TFC of 6.57 ± 0.14 mg·EQ/g [59]. *C. crepidioides* exhibited TPC of 15.34 ± 0.01 mg·GAE·g^−1^, TTC of 2.39 ± 0.01 mg·GAE·g^−1^, and TFC of 2.29 ± 0.03 mg·QEg^−1^ [62], and these values are reported to have phenol and TFCs that are approximately five times lower than the values observed with CrasMon and CrasPic (Table 4). This suggests that the two taxa have more potential in tannins, flavonoids, and overall phenols than the two plants of the same genus mentioned above. These high TPC, TFC, and TTC levels may contribute to the observed antibacterial activity (Tables 1 and 2).

Indeed, the mechanisms of action of phenolic compounds on bacterial cells have been partially attributed to the following: bacterial membrane damage, modification of cell cytoplasm pH, inhibition of virulence factors such as enzymes and toxins, and suppression of bacterial biofilm formation [63, 64]. Specifically, the antibacterial activity of flavonoids has been associated with their capacity to inhibit nucleic acid synthesis, cytoplasmic membrane function, and energy metabolism [65, 66]. Furthermore, flavonoids have been demonstrated to inhibit virulence factors and other forms of bacterial threat, including biofilm formation [67, 68]. With regard to tannins, it has been demonstrated that they inhibit bacterial growth through a number of mechanisms, including iron chelation, inhibition of cell wall synthesis, disruption of cell membranes, and inhibition of fatty acid biosynthetic pathways. Furthermore, they can act as quorum-sensing inhibitors and attenuate the gene expression of several virulence factors, including biofilms, enzymes, adhesins, motility, and toxins [66, 69, 70].

The TA content was determined to be 14.2% and 15.8% (on a dry weight basis), with the ash insoluble in hydrochloric acid exhibiting a range of 4.04%–5.03% (Table 6). The results demonstrated that CrasMon and CrasPic exhibited no significant difference.

In the genus Crassocephalum, only two species, *C. crepidioides*, have been documented to demonstrate LOD and TA. The former has a LOD of 10.6 ± 0.22% and a TA of 13.15 ± 0.18% [71]. The ash rates of this taxon are lower than those of our two taxa, CrasMon and CrasPic, while their desiccation loss rates are approximately 10 times lower. The same is true of *Bidens pilosa* L. (TA: 12.47%, LOD: 4.1%) [72] and *Securidaca longepedunculata* Fresen (TA: 6.21 ± 0.23%; LOD: 10.38 ± 0.43%) [73], two plants utilized in traditional Congolese medicine for the treatment of bacterial infections. The moisture content of a plant provides insight into its capacity for conservation, as a low water content is unfavorable for oxidation reactions, fermentation, and mold development. These phenomena have the potential to alter the quality of the active ingredient when stored for an extended period of time [74, 75]. The low levels observed in the two taxa examined in this study, CrasPic and Cras Mon, would result in a more pronounced shelf life than that of *C. crepidioides*, as well as the taxa *Bidens pilosa* and *Securidaca longepedunculata*. In terms of ash totals, these provide insight into the plant's mineral potential. Prior research has indicated that minerals can influence the expression of antibacterial activity [76, 77]. This is demonstrated by their capacity to reduce the intracellular pH of bacteria, thereby inactivating vital biochemical reactions. Consequently, the notable ash concentrations observed in the two taxa (Table 6), indicative of substantial mineral content in the plant, may contribute to the observed antibacterial activity.

### 4.4. Correlation Between Antioxidant Activity, Antibacterial Activity, and Total Phenols and Flavonoids in CrasPic and CrasMon

In this study, a positive linear correlation was observed between the antibacterial activity against *Escherichia coli,* and *Salmonella typhi* of the methanolic extract of CrasPic and CrasMon and the content of total phenols, flavonoids, and tannins (*R* > 0.9; Figure 2). This suggests that increasing the content of phenols, flavonoids, and total tannins enhances the antibacterial effect of the plants studied, indicating their involvement in the expression of antibacterial activity against these germs. The contribution of phenols, particularly flavonoids, and tannins, to the expression of antibacterial activity, has been previously reported for certain taxa. These include the leaves, stem barks, and root barks of *Papaver rhoeas* L [78], or the stem barks, leaves, and fruits of *Rhus verniciflua* Stokes, synonym of *Toxicodendron vernicifluum* (Stokes) FA Barkley [79]. Additionally, the leaves of *Allium tripedale* Trautv (*Nectaroscordum tripedale)* and *Crocus sativus* L [80], the leaves, and stem barks of *Zeravschania membranacea* (Boiss.) Pimenov, and *Ferulago angulata* (Schltdl) Boiss [81], and the leaves of *Papaver rhoeas* L [78] have been shown to contain phenols, notably, flavonoids and tannins, which contribute to the expression of antibacterial activity.

A positive linear correlation between antioxidant and antibacterial activities on *E. coli* and *S. typhi* was observed in the aerial parts of these two taxa, with an R-value greater than 0.92 (Figure 3). This suggests that antibacterial activity increases with the content of antiradical compounds. This underscores the significance of the role of free radical scavengers in modulating bacterial activity. This phenomenon has also been observed in specific taxa for which a correlation between their free radical scavenging compounds, and antibacterial activity has been established. This is particularly evident in the case of phenols in *Retama raetam* (Forssk) Webb. & Berthel [82] and in *Matricaria pubescens* (Desf) Sch Bip synonym of *Otoglyphis pubescens* (Desf) Pomel [83]. A multitude of phenolic compounds exhibits both antioxidant and antibacterial properties, likely due to their capacity to scavenge oxygenated compounds, which leads to their elimination from bacterial cells, and subsequent inhibition of numerous vital oxygen-based processes within the bacteria, ultimately resulting in cell death [36, 44, 84]. This may explain why, in some studies, such as the present one, a positive correlation is observed between antioxidant and antibacterial activities [45, 85]. Nevertheless, other phytochemical groups may also be responsible for antibacterial and free radical scavenging activity. This is notably the case for terpenoids, as evidenced by a previous study which demonstrated that terpineol exhibited excellent bactericidal activity against *S. aureus* strains. In contrast, carveol, citronellol, and geraniol exhibited a rapid bactericidal effect against *E. coli* [86, 87]. The findings of this study indicate that the correlation between antioxidant and antibacterial activities is likely due to the presence of phenolic compounds. Nevertheless, the isolation, and characterization of these compounds, coupled with these two activities, would be desirable to confirm this hypothesis.

### 4.5. Acute and Subacute Oral Toxicity of Aqueous and Ether Extracts of CrasMon and CrasPic

In addition to the absence of clinically and biologically observable toxicity, no deaths were recorded in the animals following the oral administration of ether and methanolic extracts of CrasMon and CrasPic at a dose of 5000 mg·kg^−1^ in *Cavia porcellus*. This finding suggests that the lethal dose 50 (LD_50_) of the extracts examined is higher than the administered dose. It was subsequently reported that, by the LD_50_, the extract can be considered highly toxic if the LD_50_ is equal to or less than 1 mg/kg, very harmful if the LD_50_ is between 1 mg/kg and 50 mg/kg, moderately toxic if the LD_50_ is between 50 mg/kg and 500 mg/kg, and practically nontoxic if the LD_50_ is greater than or equal to 1500 mg/kg. The LD_50_ is equal to or less than 1 mg/kg, indicating that the extracts are weakly toxic. If the LD_50_ is between 500 mg/kg and 5000 mg/kg, the extracts are considered to be moderately toxic. However, if the LD_50_ is between 5 g/kg and 15 g/kg, the extracts are practically nontoxic. Finally, if the LD_50_ is greater than or equal to 15 g/kg, the extracts are safe [88]. Therefore, the classification of CrasMon and de CrasPic as practically nontoxic is supported by these findings.

This study presents novel findings on the acute and subacute in vivo toxicity of CrasMon and CrasPic, particularly in light of the extensive existing literature that lacks any previous toxicological studies on these taxa. Prior research has investigated the subacute toxicity of particular plant taxa with documented antibacterial properties in traditional Congolese medicine. A comparison of these studies with the present study will allow for the identification of similarities and differences. This is particularly evident in the case of the aqueous extract of *Cajanus cajan* (L.) Huth leaves, for which the LD_50_ is 3715.35 mg·kg^−1^ [89]. The extracts under consideration are considerably less toxic than those previously studied. It is notable that some other species have an estimated LD_50_ exceeding 5000 mg·kg^−1^, yet clinical signs of toxicity have been documented at this dose. This is illustrated by the aqueous extract of *Cymbopogon citratus* (DC.) Stapf, which has been shown to induce sedation, fatigue, and agitation in rodents when administered in vivo [90]. The aqueous extract of *Carica papaya* L. and the methanolic extract of *Ocimum gratissimum* L. have been observed to induce steatosis [91]. Moreover, the hydroalcoholic extract of the aerial parts of *Ocimum basilicum* L. (Lamiaceae) has been demonstrated to induce disturbances in hematological parameters [92]. Additionally, instances of mortality have been documented in individuals administered the aqueous extract of *Bridelia ferruginea* Benth leaves [93], and diminished sensitivity to pain, noise, and locomotion has been observed in animals treated with the aqueous extract of *Harungana madagascariensis* Lam ex Poir stem barks [94], which contrasts with the CrasMon and CrasPic extracts examined in this study. The administration of aqueous extracts of *Nauclea latifolia* Sm. (Rubiaceae) [95], aqueous extract of Jatropha curcas L. leaves, and aqueous extract of *Alchornea cordifolia* (Schumach. & Thonn.) Müll Arg leaves [96] did not result in any observable signs with regard to the methanolic and petroleum EEs of CrasMon and CrasPic. The toxicological profile of CrasPic and CrasMon appears to be similar to that of the leaves of four other taxa utilized in traditional medicine in Katanga for the treatment of various diseases, namely, *Dialium angolense* Welw. Ex Oliv [28], *Dalbergia katangensis* Lecheneaud [22], *Ochna schweinfurthiana* F Hoffm (Ochnaceae), and *Rothmannia engleriana* (K. Schum.) Keay (Rubiaceae) [25].

The various observations made during the evaluation of acute and subacute toxicity, notably regarding variations in body weight (Figure 4(a)), organ weight (Figure 5), and biochemical markers (Table 5), demonstrate that the single oral administration of a dose of 5000 mg·kg^−1^ of ether and methanolic extracts from the leaves of these two plant species did not induce any signs of toxicity in male *Cavia porcellus* in the short term. Similarly, daily oral administration of a dose of 200 mg·kg^−1^ body weight did not induce any significant short- or medium-term toxicity in the same animal model. This constitutes the initial evidence regarding the safety of these two plants, which encourages further studies to validate their therapeutic properties. Additional studies are required to determine the effects of extracts of these two plants on female animals, chronic administration, fetal animals, pregnant animals, and their reproductive capacity, neurotoxicity, and genotoxicity. These studies are necessary to complete the safety profile of these two plants.

## 5. Conclusion

The findings of this study indicate that polar and apolar extracts derived from the CrasMon and CrasPic, whole plant, exhibit antibacterial activity and do not cause acute or subacute oral toxicity in vivo. Moreover, the study illustrates the contribution of phenolics, particularly flavonoids and tannins, in conferring the antibacterial properties of extracts derived from these two plants. This evidence could justify their use in traditional Congolese medicine, particularly against bacterial gastroenteritis. The results of this study provide a foundation for future endeavors to identify antibacterial compounds.

## Figures and Tables

**Table 1:** Inhibition zone diameter of organic extracts of *C montuosum* and *C picridifolium* at 50 *μ*g·mL^−1^.

Simple	*E. coli*	*C. jejuni*	*S. aureus*	*S. sonnei*	*S. typhi*
CIP	28.0 ± 1.2	26.1 ± 0.4	18.1 ± 0.2	25.0 ± 0.1	25.1 ± 0.4
CrasMonM	18.2 ± 0.2	10.7 ± 0.1	14.0 ± 0.2	09.0 ± 0.2	10.2 ± 1.2
CrasMonE	14.1 ± 0.2	16.1 ± 0.2	07.8 ± 1.2	08.1 ± 0.2	12.1 ± 0.2
CrasPicM	22.9 ± 0.1	10.0 ± 0.1	21.0 ± 0.2	12.2 ± 0.2	21.3 ± 0.2
CrasPicE	18.1 ± 0.2	08.8 ± 0.2	08.0 ± 0.1	15.1 ± 0.2	17.1 ± 0.2

*Note:* Results expressed as mean ± standard deviation with *n* = 3; ciprofloxacin (CIP) was used as a positive control (DZI: 18–28 mm), and the dilution solvent (5%) from each extract was used as negative control. No zone of inhibition was observed in the negative control.

Abbreviations: CrasMonE, *Crassocephalum montuosum* petroleum ether extract; CrasMonM, *Crassocephalum montuosum* methanolic extract; CrasPicE, *Crassocephalum picridifolium* petroleum ether extract; CrasPicM, *Crassocephalum picridifolium* methanolic extract.

**Table 2:** Minimum inhibitory concentration (MIC in μg·mL^−1^) and minimum bactericidal concentration (MBC in μg/mL) of organic extracts of *C montuosum* and *C picridifolium*.

Extracts	Bacteria	MIC	MBC	MBC/MIC	Effects
CIP	*E. coli*	1.95	1.95	1	BC
	*C. jejuni*	3.90	3.90	1	BC
	*S. aureus*	3.90	3.90	1	BC
	*S. sonnei*	3.90	3.90	1	BC
	*S. typhi*	1.95	1.95	1	BC

CrasMon M	*E. coli*	15.63	15.63	1	BC
	*C. jejuni*	125	125	1	BC
	*S. aureus*	31.25	31.25	1	BC
	*S. sonnei*	500.00	500.00	1	BC
	*S. typhi*	125.00	125.00	1	BC

CrasMon E	*E. coli*	62.50	500.00	8	BS
	*C. jejuni*	31.25	125.00	4	BS
	*S. aureus*	500.00	500.00	1	BC
	*S. sonnei*	500.00	500.00	1	BC
	*S. typhi*	62.50	250.00	4	BS

CrasPicM	*E. coli*	15.63	15.63	1	BC
	*C. jejuni*	62.50	62.50	1	BC
	*S. aureus*	15.63	15.63	1	BC
	*S. sonnei*	62.50	62.50	1	BC
	*S. typhi*	15.625	15.625	1	BC

CrasPicE	*E. coli*	15.63	15.63	1	BC
	*C. jejuni*	500.00	500.00	1	BC
	*S. aureus*	500.00	500.00	1	BC
	*S sonnei*	31.25	31.25	1	BC
	*S typhi*	32.25	32.25	1	BC

*Note:* Ciprofloxacin was used as a positive control (MIC < 5 *μ*g/mL), while culture medium and 5% dilution solvents (methanol or petroleum ether) were used as negative controls. No antimicrobial activity was observed in the latter.

Abbreviations: BC, bactericide; BS, bacteriostatic; CIP, ciprofloxacin; CrasMonE, *Crassocephalum montuosum* petroleum ether extract; CrasMonM, *Crassocephalum montuosum* methanolic extract; CrasPicE, *Crassocephalum picridifolium* petroleum ether extract; CrasPicM, *Crassocephalum picridifolium* methanolic extract.

**Table 3:** Phytochemical groups of antibacterial interest identified in the aerial parts of CrasPic and CrasMon.

Phytochemicals group	CrasMon	CrasPic
Alkaloids	−	−
Anthraquinones	−	+
Coumarins	+	+
Flavonoids	+	+
Polyphenols	+	+
Saponins	+	+
Steroids	−	+
Tannins	+	+
Terpenoids	+	+

*Note:* present, +; absent, −; screening performed on powders of whole plant.

Abbreviations: CrasMon, *Crassocephalum montuosum*; CrasPic, Crassocephalum picridifolium.

**Table 4:** Content of total phenols, total flavonoids, and total tannins in extracts from the whole plant of *C montuosum* and *C picridifolium*.

Extract	Total phenols (mg·GAE·g^−1^)	Total flavonoids (mg·QE·g^−1^)	Total tannins (mg·GAE·g^−1^)
CrasMon M	223.3 ± 1.9	50.48 ± 0.44	85.0 ± 5.0
CrasMon E	275.8 ± 1.9^a^	53.33 ± 0.43	106.7 ± 7.6^a^
CrasPic M	300.8 ± 3.1^a^	56.34 ± 0.35^a^	155.0 ± 5.0^a^
CrasPic E	253.3 ± 3.1	52.48 ± 0.44	138.3 ± 7.6^a^

*Note:* ANOVA test comparison with CrasMonM (($\overset{¯}{X}$) ± *S*, *n* = 3).

Abbreviations: CrasMonE, ether extract of *Crassocephalum montuosum*; CrasMonM, methanolic extract of *Crassocephalum montuosum*; CrasPicE, ether extract of *Crassocephalum picridifolium*; CrasPicM, methanolic extract of *Crassocephalum picridifolium*; GAE, gallic acid equivalent, QE, quercetin equivalent.

^a^If *p* < 0.01.

**Table 5:** Biochemical and hematological parameters assessed in the evaluation of the subacute toxicity of organic extracts of CrasPic and CrasMon.

	Control	CrasMonM	CrasMonE	CrasPicM	CrasPicE
AST(UI/L)	30.7 ± 0.21	30.8 ± 0.11	30.3 ± 0.12	30.9 ± 0.13	31.1 ± 0.21
ALT(UI/L)	31.5 ± 0.22	31.6 ± 0.21	31.2 ± 0.14	31.7 ± 0.17	31.6 ± 0.13
ALP (UI/L)	54.4 ± 0.31	54.8 ± 0.21	54.5 ± 0.12	54.6 ± 0.18	54.7 ± 0.21
Urea (mg/dL)	23.1 ± 0.11	23.3 ± 0.12	23.1 ± 0.13	23.2 ± 0.11	23.2 ± 0.17
Creatinine (mg/dL)	1.21 ± 0.14	1.22 ± 0.11	1.21 ± 0.15	1.23 ± 0.16	1.25 ± 0.13
WBC (×10^3^/mm^3^)	7.7 ± 0.22	7.7 ± 0.14	7.6 ± 0.23	7.8 ± 0.24	7.8 ± 0.26
RBC (×10^6^/mm^3^)	5.6 ± 0.41	5.6 ± 0.31	5.7 ± 0.33	5.6 ± 0.61	5.7 ± 0.37
HTC (%)	44.3 ± 0.51	44.6 ± 0.53	44.3 ± 0.36	44.7 ± 0.26	44.7 ± 0.24
SV (mm/h)	3.5 ± 0.32	3.6 ± 0.54	3.5 ± 0.12	3.5 ± 0.23	3.5 ± 0.35

*Note:* (($\overset{¯}{X}$) ± *S*, *n* = 3).

Abbreviations: ALP, alkaline phosphatase; ALT, alanine aminotransferase; AST, aspartate aminotransferase; CrasMonE, *Crassocephalum montuosum* petroleum ether extract; CrasMonM, *Crassocephalum montuosum* methanolic extract; CrasPicE, *Crassocephalum picridifolium* petroleum ether extract; CrasPicM, *Crassocephalum picridifolium* methanolic extract; HTC, hematocrit; RBC, red blood cell; SV, sedimentation rate; WBC, white blood cell.

**Table 6:** Physicochemical parameters of powders from the aerial parts of *C montuosum* and *C picridifolium*.

Parameter	Unit	CrasMon	CrasPic
Total ash	%	15.78 ± 1.92	14.24 ± 0.73
Hydrochloric ash	%	04.04 ± 0.37	05.03 ± 0.24
Loss on drying	%	1.02 ± 0.16	00.96 ± 0.21
Swelling index	mL	06.2 ± 0.55	04.91 ± 0.12
pH of a 10% aqueous solution (w/v)	NA	05.4 ± 0.30	06.71 ± 0.40

Abbreviation: NA, nonapplicable.

## Data Availability

All data are available upon request to the corresponding author.

## Nomenclature

AA: Ascorbic acid
AAB: Antibacterial activity
AOA: Antioxidant activity
-: Absent
ALP: Alkaline phosphatase
ALT: Alanine aminotransferase
AST: Aspartate aminotransferase
BC: Bactericide
BS: Bacteriostatic
CFU: Colony-forming units
CIP: Ciprofloxacin
NC: Negative control
CrasMon: *Crassocephalum montuosum*
CrasMonM: Methanolic extract of *Crassocephalum montuosum*
CrasPic: Crassocephalum picridifolium
DPPH: 1,1-diphenyl-2-picrylhydrazyl radical
DZI: Diameter of the zone of inhibition
EE: Petroleum ether extracts
GAE: Gallic acid equivalents
HAA: Hydrochloric acid soluble ash
HTC: Hematocrit
IC_50_: Concentration of an inhibitor where the response (or binding) is reduced by half
LOD: Loss on drying
MBC: Minimum bactericidal concentration
ME: Methanol extracts
MIC: Minimum inhibitory concentration
+: Present
QE: Quercetin equivalents
RBC: Red blood cells
SV: Sedimentation velocity
TA: Total ash
TFC: Content flavonoid content
TPC: Total phenol content
TTC: Total tannins content
WBC: White blood cells
